# Language-related eligibility criteria in UK randomised trials: a systematic review of extended research reports

**DOI:** 10.1186/s13063-026-09766-5

**Published:** 2026-05-16

**Authors:** Talia Isaacs, Andrea Vaughan, Eva Burnett, Zsófia Demjén, Marie-Anne Durand, Katie Gillies, Kamlesh Khunti, Nurulamin M. Noor, Frances Shiely, Matthew R. Sydes, Shaun Treweek, Katie Biggs

**Affiliations:** 1 Centre for Applied Linguistics, UCL Institute of Education, University College London, 20 Bedford Way, London, WC1H 0AL UK; 2 Unaffiliated, London, UK; 3 Unisanté, University Center for Primary Care and Public Health, Department of Ambulatory Care, University of Lausanne, Route de La Corniche 21, 1010 Lausanne, Switzerland; 4 The Dartmouth Institute for Health Policy and Clinical Practice, 1 Medical Center Drive, Lebanon, NH 03766 USA; 5 Aberdeen Centre for Evaluation, University of Aberdeen, Health Sciences Building, Foresterhill Campus, Aberdeen, AB25 2ZD UK; 6 Diabetes Research Centre, College of Life Sciences, University of Leicester, Leicester, LE1 7RH UK; 7 Department of Medicine, University of Cambridge, Hills Road, Cambridge, CB2 0QQ UK; 8 HRB Clinical Research Facility and School of Public Health, University College Cork, Cork, Ireland; 9 MRC Clinical Trials Unit at UCL, Institute of Clinical Trials and Methodology, University College London, London, UK; 10 Data for R&D, NHS England, Wellington House, Waterloo Road, London, UK; 11 School of Medicine & Population Health, University of Sheffield, Barber House, Sheffield, S10 2HQ UK

**Keywords:** Communication barriers, Cultural diversity, Depression, Eligibility determination, Ethnic minorities, Health services accessibility, Language, Minority groups, Patient selection, Randomised controlled trials as topic, Type 2 diabetes mellitus

## Abstract

**Background:**

Trial teams frequently make language-related, non-clinical eligibility decisions during recruitment. They need to ensure that patients understand the conditions and implications of trial participation and either have the necessary language skills to participate, or receive appropriate accommodations (e.g. translation or interpreting). Fair and consistent assessments are necessary to avoid unduly excluding patients, which could limit external validity and exacerbate inequalities. This study examines how trial teams make language-related eligibility decisions.

**Methods:**

We conducted a systematic review of National Institute for Health and Care Research (NIHR) research reports (2010–2022) for UK-based randomised controlled trials (RCTs) recruiting adults for two conditions that disproportionately affect ethnic minority populations: clinical depression and type 2 diabetes mellitus (T2DM). Two researchers independently screened titles and abstracts and extracted data. We analysed the communication demands of the interventions and primary outcome measures in relation to how language screening was reported, including procedures or instruments used as proxies for language-related gatekeeping.

**Results:**

We assessed 185 titles and abstracts from NIHR monographs. Thirty-two RCTs (23 depression, 9 T2DM) ultimately met our inclusion criteria. Ethnic diversity was minimal, particularly in the depression RCTs, where the median proportion of White participants was 97%. Language screening practices were inconsistent across studies and were often poorly aligned with the actual linguistic demands of the trial. Half of the included RCTs explicitly reported a language-based eligibility criterion, including 63% of trials evaluating talking therapies for depression compared to 27% of trials assessing pharmacological, device-based, or surgical interventions. Explicit and implicit language-related gatekeeping measures included the ability to complete research assessments involving language (sometimes to a prespecified score cut-point), provide informed consent, and engage in the intervention as judged by recruiters. Translation and interpreting support were mentioned in one depression study.

**Conclusions:**

This review exposes methodological practices that may impede diverse patients’ participation. Linguistic demands of the interventions and outcomes need to be considered in justifying language-related screening and accommodations. Participants’ language variables need to be disentangled from ethnicity through routine data collection. A purpose-built screening tool that is universally applied to all participants could lead to fairer, more consistent assessments.

**Trial registration:**

PROSPERO International Prospective Register of Systematic Reviews CRD42021267905. https://www.crd.york.ac.uk/prospero/display_record.php?RecordID=267905. Registered on October 21 2021.

**Supplementary Information:**

The online version contains supplementary material available at 10.1186/s13063-026-09766-5.

## Introduction

Minority ethnic groups are chronically underrepresented in UK clinical trials [1–3] despite recent efforts to improve this, notably since the COVID-19 pandemic [4]. This means that in ethnically diverse societies in the UK and other Western countries, large segments of the population that have a stake in the safety and effectiveness of the interventions being evaluated may be excluded [5]. Underrepresenting groups that are under-served [4, 6], also referred to in trials research as hard-to-reach, vulnerable, disadvantaged, seldom heard, marginalised, etc. [4], has implications for equitable access to healthcare and social inclusion and extends to trials [7]. Although recruiting and retaining members from under-served groups may be slower and more resource-intensive, this is offset by higher quality, more informative, societally relevant trial results that better serve the community [8].

Language is a common non-clinical reason for trial exclusions [9]. The reasons for this are complex. Trial recruiters often face competing pressures when determining participant eligibility in this domain. On one hand, they must ensure that prospective participants have sufficient language ability to understand the conditions and implications of trial participation: an ethical imperative [10]. On the other, language-related eligibility criteria may be explicitly stated (e.g. “must speak English”), implied, or assumed [11]. This opens the door to biased assessments that may be detached from the linguistic demands of the trial. For example, recruiters may make assumptions about language proficiency based on physical appearance before hearing a participant speak in a phenomenon known as reverse linguistic stereotyping [12]. In addition, having a perceptible foreign accent does not necessarily mean that a person is unable to communicate intelligibly and, hence, should be barred from trial participation [13].

Despite the stakes, evidence on how language ability is assessed for trial participation remains limited. An initial systematic review of 58 UK-based randomised controlled trials (RCTs) for type two diabetes mellitus (T2DM) assessing telehealth interventions found that a language eligibility criterion was reported in only half of the included studies [11]. When specified, language requirements often combined speaking, listening, reading, or writing in arbitrary ways, sometimes underspecifying or misaligning with the communication demands of the intervention. Other studies invoked a native speaker standard, although it was unclear who should determine nativeness and using which criteria [14]. A subsequent systematic review of 70 UK cardiovascular trial protocols found that only 23% mentioned language ability as a prerequisite for participation, with varied wording and no detail on how it would be assessed [15]. However, 87% referred to participants needing to be able to provide informed consent, an inherently language-mediated process, with only one study offering an alternative consent pathway. Just three protocols (4%) referred to the availability of translation, albeit without specifying which languages could be accommodated. The authors concluded that the lack of language accommodations likely hinders ethnically diverse inclusion in trials.

Although both reviews focused on language in trials, neither reported how gatekeeping decisions of participants’ eligibility on language grounds were made during recruitment. In other words, information on language operationalisation was limited, presumably because such details were absent from the included RCTs. In medical journals, word limits for articles and conventions of conciseness may constrain reporting of language screening [11]. Protocols are also unlikely to include such detail, although they do reveal what researchers considered before commencing their study (e.g. whether or not language accommodations were integral to the plan). To address this limitation, this systematic review examined whether and how trial teams report screening language in extended research reports, the most comprehensive publication type for trials. There is more space in monographs to describe operational detail (e.g. language proficiency screening) than in shorter publication formats, reducing the risk of incomplete reporting, making it suitable for our purposes. Furthermore, neither previous systematic review clearly distinguished between translation, which occurs in the written medium when text is rendered in another language, and interpreting, which occurs in the oral medium. The T2DM systematic review [11] reported them as one category in a surface way, whereas the systematic review of cardiovascular protocols [15] appeared to conflate them under the umbrella term “translation”. A more in-depth examination of these and other language accommodations could strengthen the evidence base.

In this systematic review, we focused on two conditions with higher prevalence and poorer outcomes for some ethnic minority groups: T2DM [16] and clinical depression [17], defined within the “depressive disorders” category of the Diagnostic and Statistical Manual of Mental Disorders (DSM-5) [18]. Depression presents distinctive language-related considerations, including limited cross-linguistic equivalence for key constructs (e.g. the term “depression” does not have a direct translated equivalent in many South Asian languages [19]). In addition, talking therapies, where language is integral to the intervention, may involve more elaborate language screening. T2DM was included as a comparator due to the existence of a prior systematic review examining the role of language in trials focusing on this condition as reported in journal articles [11], providing a point of comparison for the present study. It also offered a likely breadth of interventions (from biomedical to behavioural), which we anticipated would be informative when examined alongside talking therapies, particularly with respect to language screening and accommodations. We examined the following research questions:

1. What proportion of research monographs on RCTs targeting depression or T2DM report a language-based eligibility criterion?
2. How are language-related participant eligibility decisions operationalised in these RCTs (e.g. through recruiter judgments, objective tools)?
3. What language accommodations do these RCTs make?
4. Are there any differences in research questions 1–3 for RCTs where the use of language skills (i.e. listening/reading/speaking/writing) is core to:
  - The intervention (e.g. talking therapies) versus those where it is not (e.g. drug dosing trial)?
  - The primary outcome measures (e.g. self-report) versus those where it is not (e.g. biomarkers)?

## Methods

We conducted this systematic review in accordance with Cochrane’s handbook [20] and the current Preferred Reporting Items for Systematic Reviews and Meta-Analyses (PRISMA) guidelines [21], with registration and the protocol on PROSPERO: CRD 42021267905. See Additional file 1 for the PRISMA checklist.

### Patient and public involvement and engagement (PPIE)

Following the Guidance for Reporting Involvement of Patients and Public 2 (GRIPP2-SF) on the co-production of research [22], we partnered with co-author EB, a multilingual PPIE representative with lived experience as a linguistic minority in UK trials. We also collaborated with the University of Leicester’s Centre for Ethnic Health Research and the South Asian Health Foundation.

### Searches and study screening and selection

The search targeted research monographs for funded projects published by the National Institute for Health and Care Research (NIHR), the largest national funder of clinical research in Europe [23]. We included RCTs that recruited adult patients (≥18 years, consistent with legal capacity to consent to research participation) to UK-based trials targeting either clinical depression, or T2DM. Eligible studies included pilot, feasibility, and late-phase RCTs and all intervention types. Where monographs reported additional study designs within the same research programme (e.g. observational studies, non-randomised designs, nested qualitative studies, or economic evaluations), we extracted data relevant only to the RCT component. RCTs targeting type 1 or gestational diabetes, even alongside T2DM, were excluded. However, we included disease prevention trials for individuals at risk of developing either T2DM or clinical depression but without a formal diagnosis. RCTs recruiting healthcare professionals or participants lacking capacity to consent, including those enrolled under emergency consent procedures, were excluded. If any subset of participants within a trial could not provide their own consent or there was any ambiguity, the study was excluded.

The second author conducted the initial search on 17-Nov-2021 after consulting a subject librarian and ran a repeat search on 11-Feb-2022 using a now retired version of the NIHR Journals Library database (https://www.journalslibrary.nihr.ac.uk/). Notably, NIHR Journals Library launched a new website in 2024, using a different underlying search engine that applies varied weightings to different fields (H. Nolan, personal communication, 10-July-2025). As a result, the original search is no longer replicable. NIHR Libraries is not a standard search engine. Boolean operators do not function as expected, there is no option to filter studies by participants’ age, for example, and trials are not a distinct search category. Using “trial” as a keyword alongside the condition was too restrictive; therefore, manual screening was performed after the searches. We explored identifying NIHR monographs using MEDLINE; however, only one NIHR journal, *Health Technology Assessment* (HTA), was indexed on other databases, making the NIHR Journals Library the only search tool that covered monographs for all NIHR-funded programmes. We first searched for “depression” then repeated the procedure for “diabetes” using the following fixed-choice parameters:

- Research type: primary research
- Research status: published
- Report published before: Feb-2022
- Report published after: Jan-2010

The timeframe was selected to capture language screening and reporting over a >10-year period, although some NIHR funding schemes commenced after 2010. We acknowledge the time lag since the search and the increased emphasis on diversity and inclusion in recent years, driven, in part, by NIHR initiatives [4, 6]. However, given that NIHR’s mandated inclusion requirements as a condition for funding came into effect on 27-Nov-2024 [24] and Consolidated Standards of Reporting Trials (CONSORT) only listed language alongside other demographic variables in its accompanying guidance in 2025 [25], not in earlier versions, RCTs published between Feb-2022 (review end date) until before those changes came into effect would likely show similar findings to those observed in the current review. Updating the search at the time of writing using a substantially altered interface, while desirable, would introduce methodological inconsistency and compromise the comparability of the evidence base and, therefore, was not an option. The NIHR Journals Library closed for new submissions on 30-April-2025 and submission to NIHR journals in monograph format for funded research is no longer required. Therefore, this review offers insight into the most comprehensive reporting practices in stand-alone publications prior to the discontinuation of this format.

Two of three researchers (the first, second, or last author) independently conducted title, abstract, and full-text screening against the eligibility criteria, resolving discrepancies through discussion. Data extraction was dual-coded and verified by a third researcher. A data extraction spreadsheet, informed by Cochrane guidelines [20], captured details on RCT designs, procedures, interventions, and participant characteristics (see Additional file 2). In line with our research questions, we extracted variables on how language eligibility is framed and operationalised, the language demands of the intervention and outcome measures, and any accommodations provided.

### Data analysis and narrative synthesis

Following data extraction (see Additional file 3), we identified all eligibility statements that explicitly referenced language, implied its use, or where language may have been a factor. One researcher classified statements as either directly referring to language, or using language as a proxy for eligibility (see Additional file 4). After verifying this categorisation, a second researcher grouped semantically similar eligibility statements together in an iterative process. A third researcher reviewed groupings and codes, resolving ambiguous cases through discussion, and contributed to the development of researcher-articulated categories. We treated similar eligibility statements in different parts of the monograph (e.g. *Scientific Summary* and *Methods*) as separate statements to examine consistency and semantic differences. We did not conduct a meta-analysis due to expected heterogeneity.

For each language-related eligibility criterion, we coded whether there was a presence or absence of three elements: (1) language and/or communication skills, (2) RCT tasks (e.g. research assessments, intervention, providing informed consent), and (3) how the language skills or abilities required for participation would be assessed. Each criterion was then coded for either (a) positive framing, emphasising what participants can or need to be able to do, or (b) negative framing, emphasising deficits, including inability or unwillingness to meet the requirements. Our review centred on language screening during recruitment rather than on intervention effects on health outcomes. Thus, we focused on the nature and validity of language-related screening procedures rather than quality assessments of included studies, as specified in the protocol.

We also coded the language demands of the intervention and primary outcome measure to explore whether the presence or absence of language-related eligibility criteria varied according to their language intensity (see Additional file 4). Finally, we examined whether and how language-related eligibility criteria aligned with the communication demands of the intervention and primary outcomes and considered possible sources of construct-irrelevant variance, which is when extraneous variables threaten the validity of the assessment [26].

## Results

### Study selection

**Fig. 1:**
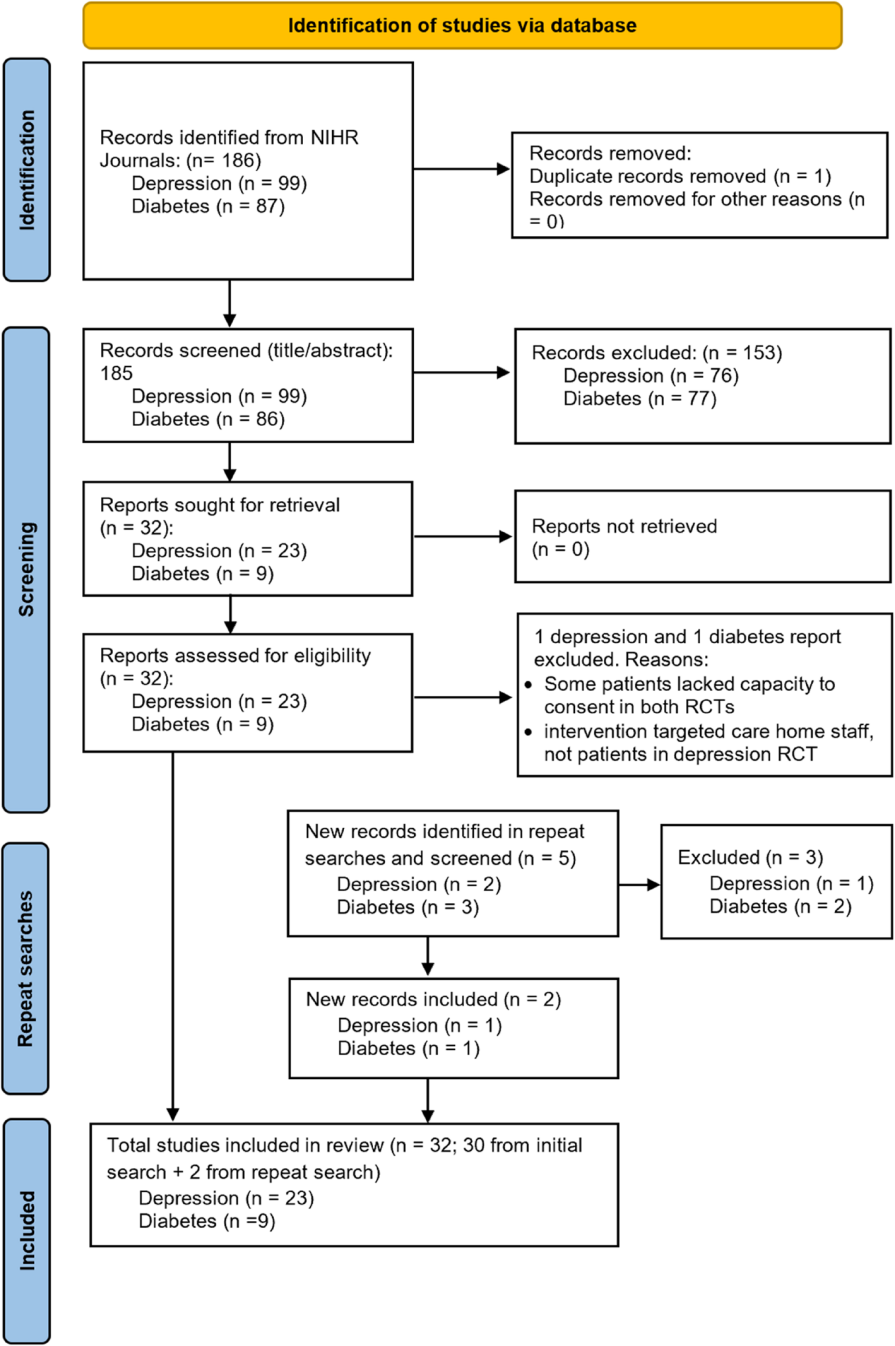
PRISMA flow diagram summarising the selection process for eligible RCTs

The search yielded 185 records after removing duplicates (see PRISMA flow diagram in Fig. 1). Title and abstract screening resulted in the exclusion of 153 records. Two more were excluded because participants lacked capacity to consent and one study recruited staff rather than patients. The full list of excluded studies and reasons for the exclusions are shown in Additional file 5. Following the repeat search, two additional records met the inclusion criteria, bringing the total to 32 NIHR monographs: 23 targeting depression [27–49] and 9 targeting T2DM [50–58].

### Methodological characteristics of included RCTs

Table 1 summarises methodological characteristics of the 32 included RCTs. Over two-thirds (22/32) were funded through NIHR’s HTA programme, with the remainder funded by *Efficacy & Mechanism Evaluation* (8/32), *Health & Social Delivery Research* (1/32), and *Public Health Research* (1/32). Over 85% (28/32) of included RCTs were individually randomised, with four cluster RCTs [35, 44, 50, 58]. There was a mix of pilot (2/32), feasibility RCTs (5/32; including one that used both terms), and definitive (26/32) trials and the majority (25/32) self-described as “pragmatic” trials. All but one definitive trial [58] and two feasibility studies [45, 52] were multicentre trials (≥3 sites; 29/32) and all were two-armed, except for 3/32 studies with three arms [33, 56, 57].

**Table 1:** Trial design and Population, Intervention, Comparison, Outcome (PICO)

Surname, first author	Year	NIHR journal	Trial design	Population	Intervention	Control	Primary outcome measure
Depression trials							
Sharp [27]	2010	Health Technology Assessment (HTA)	Pragmatic, multicentre, two-arm, individually randomised controlled trial (RCT)	Postnatal depression	Health visitor non-directive counselling (listening visits)	Antidepressants as prescribed by general practitioner (GP)—treatment as usual (TAU)	Edinburgh Postnatal Depression Scale (EPDS)
Chalder [28]	2012	HTA	Pragmatic, multicentre, two-arm, individually randomised RCT	Mild or moderate depression	Physical activity as a treatment for depression (TREAD)	Matched placebo	Beck Depression Inventory (BDI)
Bedson [29]	2014	HTA	Pragmatic, multicentre (3 centres), two-arm, individually randomised RCT	Depression	Folic acid (+ usual anti-depressant medication)	Matched placebo	Beck Depression Inventory-II (BDI-II)
Wiles [30]	2014	HTA	Pragmatic, multicentre, two-arm, individually randomised RCT	Treatment-resistant depression	Cognitive behavioural therapy (CBT) (+ usual care)	TAU	BDI-II
Ferrier [31]	2015	Efficacy and Mechanism Evaluation (EME)	Multicentre, two-arm, individually randomised RCT	Depression	Drug: metyrapone	Matched placebo	Montgomery–Åsberg Depression Rating Scale (MADRS)
Kuyken [32]	2015	HTA	Pragmatic, multicentre, two-arm, individually randomised RCT	Depression	Mindfulness-based cognitive therapy with support to taper (MBCT-TS)	Maintenance antidepressants	Longitudinal Interval Follow-up Evaluation (LIFE), a form of Structured Clinical Interview for DSM Disorders (SCID)
Littlewood [33]	2015	HTA	Pragmatic, multicentre, three-arm, individually randomised RCT	Depression	Comparing paid vs free computerised CBT	TAU	Patient Health Questionnaire-9 (PHQ-9)
Brabyn [34]	2016	HTA	Pragmatic, multicentre, two-arm, individually randomised RCT	Depression	MoodGym app therapy	MoodGym with a free help line but without the regular telephone calls	PHQ-9
Richards [35]	2016	EME	Pragmatic, multicentre, two-arm, cluster RCT	Depression	Collaborative care	TAU	PHQ-9
Anderson [36]	2017	EME	Multicentre, two-arm, individually randomised RCT	Depression	Electroconvulsive therapy	Intravenous saline—0.9% sodium chloride solution	Hopkins Verbal Learning Task—Revised (HVLT-R)
Bosanquet [37]	2017	HTA	Pragmatic, multicentre, two-arm, individually randomised RCT	Major depression	Collaborative care	TAU	PHQ-9
Gabbay [38]	2017	HTA	Pragmatic, multicentre, two-arm, individually randomised RCT	Depression (with worries about debt)	Collaborative care	TAU and two debt advice leaflets	BDI-II
Lewis [39]	2017	HTA	Pragmatic, multicentre, two-arm, individually randomised RCT	Subthreshold depression	Collaborative care	TAU	PHQ-9
Richards [40]	2017	HTA	Pragmatic, multicentre, two-arm, individually randomised RCT	Depression—major depressive disorder	Behavioural activation (BA) and CBT (two active treatment arms)	*Not applicable—two treatment arms*	PHQ-9
Jahoda [41]	2018	HTA	Multicentre, two-arm, individually randomised RCT	Depression (in adults with learning disabilities)	“BeatIt”—BA	Active control—“StepUp”: guided self-help intervention	Glasgow Depression Scale for people with a Learning Disability (GDS-LD)
Kessler [42]	2018	HTA	Pragmatic, multicentre, two-arm, individually randomised RCT	Depression	Mirtazapine	Matched placebo	BDI-II
Lynch [43]	2018	EME	Multicentre, two-arm, individually randomised RCT	Major depressive disorder	Radically open dialectical behaviour therapy (RO DBT)	TAU	Hamilton Rating Scale for Depression (HRSD)
Richards [44]	2018	HTA	Pragmatic, multicentre, two-arm, cluster randomised controlled pilot trial	Depression with coronary heart disease	Enhanced psychological care	TAU	No primary outcome identified as it was a pilot trial. Clinical outcomes collected: BDI-II, BAI, EuroQol 5 Dimensions (EQ-5D), EuroQol 5 Dimensions Five-Level Version (EQ-5D-5L), EuroQol Visual Analogue Scale (EQ-5D VAS), Heart Quality of Life Questionnaire (HeartQoL), Behavioural Activation for Depression Scale—Short Form (BADS-SF)
Burroughs [45]	2019	Health and Social Care Delivery Research	Feasibility RCT—pragmatic, multicentre, two-arm, individually randomised RCT	Depression and anxiety (older adults)	Non-Traditional providers to support the management of Elderly People with Anxiety and Depression (NOTEPAD) intervention	TAU	Clinical Interview Schedule-Revised (CIS-R)
Serfaty [46]	2019	HTA	Pragmatic, multicentre, two-arm, individually randomised RCT	Depression in advanced cancer	CBT	TAU	BDI-II
Thomas [47]	2019	HTA	Feasibility RCT—pragmatic, multicentre, two-arm, individually randomised RCT	Depression after stroke	BA	TAU	PHQ-9
Ali [48]	2021	Public Health Research (PHR)	Pragmatic, 2 centre, two-arm, individually randomised pilot RCT	Depression in individuals with intellectual disability	1-to-1 volunteer befriending	TAU and a copy of the activities booklet	GDS-LD
Duffy [49]	2021	HTA	Pragmatic, multicentre, two-arm, individually randomised RCT	Depression relapse (in patients taking antidepressants, but well enough to consider stopping medication)	Tapering (discontinuing) antidepressants with placebo tablets	TAU—remain on current medication	CIS-R
Diabetes trials							
Simmons [50]	2016	HTA	Pragmatic, multicentre, two-arm, cluster RCT	People with type 2 diabetes mellitus (T2DM)	Medication plus lifestyle changes	TAU, GPs were provided with the diagnostic test results	Composite of first cardiovascular event, including cardiovascular mortality, cardiovascular morbidity (non-fatal myocardial infarction and non-fatal stroke), revascularisation, and non-traumatic amputation
Griffin [51]	2018	HTA	Feasibility RCT—pragmatic, multicentre, two-arm, individually randomised RCT	Non-diabetic hyperglycaemia	Slow-release metformin	Placebo	No clinical primary specified as primary outcomes are feasibility Clinical outcomes collected: (1) cardiovascular events, (2) cancer events, and (3) other events including diabetes
House [52]	2018	HTA	Feasibility RCT—multicentre, two-arm, individually randomised RCT	People with T2DM and a learning disability	OK Diabetes, supported self-management, plus TAU	Easy Read booklet, TAU	Primary trial outcomes were feasibility outcomes, but two candidate primary clinical outcomes were collected: glycated haemoglobin (HbA1c) and body mass index (BMI)
Sivaprasad [53]	2018	EME	Pragmatic, multicentre, two-arm, individually randomised RCT	Proliferative diabetic retinopathy	Intravitreal aflibercept	Laser eye therapy—panretinal laser photocoagulation	Best corrected visual acuity measured in Early Treatment Diabetic Retinopathy Study (ETDRS) letter score at 4 m
Sivaprasad [54]	2019	EME	Multicentre, two-arm, individually randomised RCT	Diabetic macular oedema (MO)	Light masks during sleep for 24 months	Placebo (sham) mask that did not emit light	Optical coherence tomography (OCT)
Ruban [55]	2020	EME	Multicentre, two-arm, individually randomised RCT	Obesity with T2DM	Compare efficacy of medical therapy with/out gastric bypass	No liner, just diet help for diabetes control	HbA1c
Hykin [56]	2021	HTA	Pragmatic, multicentre, three-arm, individually randomised RCT	MO due to central retinal vein occlusion (CRVO)	Aflibercept (2.0 mg/0.05 ml) and bevacizumab (1.25 mg/0.05 ml)	Ranibizumab—usual care/3 arm active treatment	ETDRS letter score
Khunti [57]	2021	HTA	Pragmatic, multicentre, three-arm, individually randomised RCT	People at risk of T2DM	Walking Away, or Walking Away Plus	Advice leaflet	Change in steps per day
Miras [58]	2021	EME	Pragmatic, multicentre, two-arm, cluster RCT	T2DM and obesity	Long biliopancreatic limb Roux-en-Y gastric bypass	50 cm (standard limb) Roux-en-Y gastric bypass	Postprandial peak of active glucagon-like peptide-1 receptor agonists (GLP-1) concentration

Abbreviations: *BA* behavioural activation, *BADS-SF* Behavioural Activation for Depression Scale—Short Form, *BAI* Beck Anxiety Inventory, *BDI* Beck Depression Inventory, *BDI-II* Beck Depression Inventory-II, *CBT* cognitive behavioural therapy, *CIS-R* Clinical Interview Schedule—Revised, *ETDRS* Early Treatment Diabetic Retinopathy Study, *EME* Efficacy and Mechanism Evaluation, *EQ-5D VAS* EuroQol Visual Analogue Scale, *EQ-5D* EuroQol 5 Dimensions, *EQ-5D-5L* EuroQol 5 Dimensions, Five-Level Version, *GP* general practitioner, *GDS-LD* Glasgow Depression Scale for People with a Learning Disability, *HRSD* Hamilton Rating Scale for Depression, *HTA* Health Technology Assessment, *HVLT-R* Hopkins Verbal Learning Task—Revised, *MO* macular oedema, *MBCT-TS* Mindfulness-Based Cognitive Therapy with Support to Taper, *NIHR* National Institute for Health and Social Care Research, *OCT* optical coherence tomography, *PRP* panretinal photocoagulation, *PHQ-9* Patient Health Questionnaire-9, *PICO* Population, Intervention, Comparison, Outcome, *PHR* Public Health Research, *RCT* randomised controlled trial, *SCID* Structured Clinical Interview for DSM Disorders, *TAU* treatment as usual, *T2DM* type 2 diabetes mellitus

### Sample characteristics of included RCTs

Table 2 summarises participant characteristics in the included studies. All trials recruited both males and females except for one study on postnatal depression in women [27]. Among depression RCTs, marital status was the most reported sociodemographic variable (16/23), followed by employment status or occupation (12/23) and education or qualifications (12/23). Financial indicators appeared in only 2/23 studies [30, 43], social class in two others (2/23) [32, 45], and 3/23 studies reported no socioeconomic variables [31, 44, 47]. T2DM RCTs reported such characteristics less frequently. One reported employment status [50] while another, the only ethnically targeted trial in the dataset, reported social deprivation, occupation, education level, marital status, and internet access [57]. This study set a recruitment quota of one-quarter South Asian participants and adopted lower age thresholds for this group due to higher T2DM susceptibility. Only one other T2DM RCT [51] referenced ethnic diversity as a research objective, focusing on recruitment feasibility. Both studies examined interventions for participants at risk of developing T2DM but neither elaborated a recruitment/retention strategy for ethnic minority participants. No other RCT aimed for ethnically diverse or representative samples as part of their research objectives, although some depression studies mentioned diversity in embedded qualitative components.

**Table 2:** Sample characteristics for included studies

First author	Year	Total recruited	Age of sample	Sex/gender	Ethnicity	Socioeconomic variables collected
Depression trials						
Sharp [27]	2010	254	Mean = 31.4; SD = 5.6	Female = 254/254	White = 196/252; Black = 29/252; Asian = 13/252; other = 14/252	Marital status; living arrangements; number of children; employment status; occupation; level of education
*Chalder [28]	2012	361	Calculated mean = 39.9	Male = 122/361; female = 239/361	White = 336/361	Marital status; employment status; homeowner; level of education
Bedson [29]	2014	475	Mean = 45; SD = 13; range = 19–81	Male = 160/440; female = 280/440	White = 427/440; other = 5/440; not stated/missing = 8/440	Marital status; employment status
Wiles [30]	2014	469	Mean = 49.6; SD = 11.7	Male = 130/469; female = 339/469	White = 459/469	Marital status; employment status; level of education; financial difficulty
*Ferrier [31]	2015	165	Calculated mean = 46.4	Male = 66/165; female = 99/165	White = 157/165; other = 8/165	–
*Kuyken [32]	2015	424	Calculated mean = 49.5	Male = 99/424; female = 325/424	White = 420/424	Marital status; level of education; social class (ONS grading)
*Littlewood [33]	2015	691	Calculated mean = 39.9	Male = 229/691; female = 462/691	White—British = 657/691; White—Irish = 1/691; any other White = 16/691; mixed WB Caribbean = 3/691; mixed WB African = 1/691; other mixed = 1/691; Indian = 2/691; Pakistani = 1/691; Chinese = 4/691; other = 5/691	Marital status; level of education; employment status; occupation
Brabyn [34]	2016	369	Mean = 40.6; SD = 13.8; median = 40.6; range = 18.2–77.1	Male = 131/369; female = 238/369	White British = 347/369; (multiple groups reported)	Level of education; employment status; occupation
Richards [35]	2016	581	Mean = 44.8; SD = 13.3; range = 17–82	Male = 163/581; female = 418/581	White British = 494/581; other = 87/581	Level of education; employment status; marital status
*Anderson [36]	2017	79	Calculated mean = 54.6	Male = 35/79; female = 44/79	White = 66/79	Marital status
*Bosanquet [37	2017	485	Calculated mean = 72.2	Male = 183/484; female = 301/484	White = 474/484; Asian = 1/484; Black = 1/484; other = 5/484	Level of education
Gabbay [38]	2017	61	Mean = 46; SD = 12.8; range = 21–79	Male = 26/61; female = 35/61	White = 59/61; other = 2/61	Marital status; occupation
*Lewis [39]	2017	344	Calculated mean = 77.3	Male = 298/702; female = 407/702	White = 698/702; Asian = 2/702; Black = 2/702; other = 1/702	Level of education
Richards [40]	2017	440	Mean = 43.5; SD = 14.1; range = 18–84	Male = 150/440; female = 290/440	White British = 401/440; other = 39/440	Marital status; level of education
*Jahoda [41]	2018	161	Calculated mean = 40.2	Male = 76/161; female = 85/161	White = 156/161; other = 3/161; unknown = 2/161	Marital status
*Kessler [42]	2018	480	Calculated mean = 53.2	Male = 148/480; female = 332/480	White = 468/480; mixed = 8/480; Asian = 2/480; other = 2/480	Marital status; employment status
*Lynch [43]	2018	162	Calculated mean = 47.4	Male = 86/250; female = 164/250	White British = 222/250; White other = 10/250; other = 6/250	Income; level of education; marital status
*Richards [44]	2018	29	Calculated mean = 65.5	Male = 15/29; female = 14/29	White = 29/29	–
Burroughs [45]	2019	38	Median = 71; IQR = 68–76	Male = 16/38; female = 22/38	British = 38/38	Marital status; social class/employment level
Serfaty [46]	2019	230	Mean = 59.5; SD = 11.4	Male = 78/230; female = 152/230	White = 167/230; Black = 31/230; South Asian = 13/230; other = 19/230	Employment status; level of education; marital status
Thomas [47]	2019	48	Mean = 65.6; SD = 13.6; median = 66 (55–75); range = 31–97	Male = 29/48; female = 19/48	White = 47/48; Asian = 1/48	–
Ali [48]	2021	16	Mean = 41.6; SD = 16.7	Male = 7/16; female = 9/16	White = 8/16; Asian = 4/16; Black = 1/16; mixed = 3/16	Living arrangements; multimorbidity
Duffy [49]	2021	478	Mean = 54.5	Male = 129/478; female = 349/478	White = 449/478; not White = 24/478	Marital status; employment status
Diabetes trials						
*Simmons [50]	2016	3057	Calculated mean = 60.3	Male = 1771/3057; female = 1286/3057	White = 2785/3057	Employment status
Griffin [51]	2018	249	Mean = 70	Male = 219/249; female = 30/249	White = 244/249; mixed = 0/249; Asian = 4/249; Mexican American = 1/249	–
House [52]	2018	82	Mean = 56.4	Male = 40/82; female = 42/82	White = 75/82; mixed = 2/82; Asian = 5/82	–
Sivaprasad [53]	2018	232	Mean = 51.15	Male = 155/232; female = 77/232	Collected but not reported	–
Sivaprasad [54]	2019	308	Mean = 57; SD = 11	Male = 194/308; female = 114/308	White = 194/308; Black = 36/308; Asian = 33/308; other = 4/308	–
Ruban [55]	2020	170	Mean = 51.8; SD = 8.18	Male = 92/170; female = 78/170	White = 132/170; Asian = 20/170; Black = 16/170; mixed = 2/170	–
Hykin [56]	2021	463	Mean = 69.1; SD = 13.0	Male = 265/463; female = 198/463	Collected but not reported	–
*Khunti [57]	2021	1366	Calculated mean = 59.4	Male = 693/1366; female = 673/1366	White European = 982/1366; South Asian = 305/1366; other = 79/1366	IMD; occupation; level of education; marital status; internet access
Miras [58]	2021	53	Majority middle-aged, White, female (no values specified)			–

^*^Calculated means were calculated from the means and sample size for each arm

Table 2 shows that the overwhelming majority of participants in the depression studies were White, with a median of 97% (range: 50–100%). The sole outlier, a feasibility RCT involving participants with intellectual disabilities [48], reported 50% mixed or non-White ethnicities but included only 16 participants. Whereas all depression trials reported the ethnic composition of the recruited sample, two T2DM RCTs did not report this characteristic [53, 56] and another described recruited participants as “majority White and European” without specifying proportions [58]. Of the six remaining T2DM studies, half [54, 55, 57] reported ethnically mixed samples (54–72% White), whereas the remainder exceeded 90% White participants. Some studies reported only the percentage of White participants (e.g. White British/White European), without indicating whether remaining participants were ethnic minorities or those with undisclosed or unknown ethnicity. In other cases, non-White participants were categorised as “other”.

Two depression RCTs [27, 47] and one T2DM RCT [51] that had failed to meet their objectives of ethnic representation, recruiting 98% White participants, acknowledged poor ethnic minority recruitment in relation to generalisability, with another depression RCT reporting that inadequate recording of patient ethnicity in general practitioner (GP) practices had hindered sampling on that basis. Conversely, a T2DM RCT with 37% ethnic minority participants [54] broadly highlighted the generalisability of its findings due to ethnic representativeness, although with no formal mapping to the target population.

### Narrative synthesis

Prior to reporting the main results, we summarise the language demands of the interventions and primary outcome measures. In this review, high language demands consisted of one-to-one interventions in-person or over the phone (e.g. talking therapies). These interventions required comprehension, interaction, and oral expression during one-to-one real-time communication. Eight depression RCTs [27, 30, 33, 34, 38, 40, 43, 46] but no diabetes RCTs were in this category. Medium language demand interventions included group counselling or educational sessions (e.g. lifestyle advice) and behavioural activation (BA), which one study described as “less dependent on verbal communication” than talking therapies [41], focusing on practical steps or counsellor-facilitated discussion rather than in-depth verbal expression of complex experiences. Ten depression studies were classified as medium demand [28, 32, 35, 37, 39, 41, 44, 45, 47], six of which included participants with mild/moderate intellectual or learning disabilities [41, 48], cognitive impairment [37, 39], or post-stroke aphasia [47]. Three diabetes studies involving behavioural interventions for lifestyle change [50, 57] or education around self-management [52] were also in this category. Low language demand interventions included pharmacological, surgical, or device-based treatments, where language was not integral to the intervention, although patients may have needed to follow instructions (e.g. regarding dosing). Five depression RCTs [29, 31, 36, 42, 49] and the 6/9 remaining diabetes RCTs [51, 53–56, 58] were coded as low demand interventions (see Additional file 3).

All but one depression trial (22/23) used participant self-report data as the primary outcome measure, typically assessing depression severity. Three RCTs administered such measures verbally via structured clinical interviews [32, 45, 49], while the remainder (19/23) used written formats. The only depression study without a self-report primary outcome, an electroconvulsive therapy trial [36], which had low language demands of the intervention, used the Hopkins Verbal Learning Task—Revised (HVLT-R) to assess “delayed verbal recall”. This was the sole language-based primary outcome measure included in the review, where language was the object rather than simply the medium of assessment. In contrast, all nine included T2DM trials employed clinical or behavioural measures as primary outcomes, although 6/9 [50–52, 54, 56, 57] included self-report secondary outcomes on topics such as quality of life, medication adherence, sleep duration, and mood.

#### Prevalence of language eligibility criteria and positive or negative framing

Over half of the depression RCTs (13/23) explicitly referred to the English language as an eligibility criterion [27, 28, 33, 34, 36, 41, 43–49]. The median proportion of non-White participants was more than twice as high in these studies (5.7%) compared to the 10/23 depression RCTs that made no reference to English in the eligibility statements (2.8%) [29–32, 35, 37–40, 42]. Among the 13/23 studies specifically referencing the English language in the eligibility criteria, some referred broadly to language proficiency or communication [27, 41, 44, 47], including a study that seemingly used the term “fluent” [36] synonymously with proficiency [59]. Other RCTs specified particular language skills, though without consistent patterns across trials, including speaking [43, 48], understanding [46], understanding and reading [45], reading and writing [33, 34], “communication and comprehension” [48], and “expressive and receptive communication” [41]. In some cases, the wording was ambiguous. For example, it was unclear whether “understanding” or “comprehension” referred to listening or reading, nor which specific skills were implied by “communication” (presumably speaking or writing given its pairing with comprehension).

Ten of the 23 depression studies whose eligibility criteria referenced language specified that participants needed sufficient language skills to complete research assessments, including questionnaires and neuropsychological testing [27, 28, 36, 48, 49] or to engage in the intervention [41, 43, 44, 46, 47]. Of the remaining 13/23 depression RCTs that made no direct reference to language in the eligibility criteria, two noted elsewhere in the manuscript that researchers “assess[ed] participants’ understanding of the treatment principles” [37, 39] at baseline. A further four [29, 30, 38, 42] that omitted direct language reference in the eligibility statements included completing questionnaires or providing consent as eligibility criteria, a potential proxy for language-related gatekeeping.

By comparison, only 3/9 of the T2DM trials directly referred to language as a participant eligibility criterion. One required participants to “understand basic written and verbal English” [57] and another listed “language barrier” alongside “mental incapacity, unwillingness or inability to understand and be able to complete questionnaires” [55]. These studies recruited a relatively high proportion of non-White participants (28% and 46%, respectively). The third study specified allowing written or verbal informed consent [52]. Of the 6/9 T2DM studies without language eligibility criteria, three [53, 56, 58] did not report the ethnic composition of the recruited sample, with the latter describing only “Majority middle-aged, White, female”, two reported samples with >90% White participants [50, 51], and one recruited 37% non-White participants [54]. Additionally, three T2DM RCTs that made no direct reference to language in their eligibility criteria [51, 53, 54] cited participants’ need to be able to provide informed consent, which implies language use.

#### How language-related eligibility criteria are reported

We identified 44 eligibility statements (categories 1–7 in Additional file 4), ranging from 0 to 6 per study. We analysed the wording and meaning and noted semantic overlap within studies. For example, one depression study articulated the same language-related eligibility criterion differently in the *Methods* section (“were unable to complete self-administered questionnaires in English”) compared to the *Scientific Summary* (“could not understand questionnaires in English”). This inconsistency conflates questionnaire completion with comprehension. The depression trials with the most (3 or 4) semantically distinct language-related eligibility statements were also among the most complex and nuanced in their reporting of language screening. One targeted participants with learning disabilities [41], another recruited stroke patients [47], and the third trialled electroconvulsive therapy for major depression [36]. The stroke and electroconvulsive therapy trials were the only RCTs to articulate both the language or communication skills required to complete RCT tasks (e.g. intervention, assessments), and the standardised instruments used to evaluate them. In the second study, “communication difficulties” were assessed using the Consent Support Tool for people with aphasia [47], which incorporated gestures and drawing. In the latter [36], participants needed to achieve a Verbal Intelligence Quotient (VIQ) score cut-point alongside satisfying recruiter judgments about “fluency”. The T2DM studies with the most (two or three) language-related eligibility statements promoted physical activity in participants at risk of developing T2DM with South Asian recruitment targets [57], or nurse-supported self-management for participants with learning (i.e. educational or behavioural interventions) [52].

The first author coded 18 instances of positive framing of eligibility criteria across 11/32 RCTs (8 depression, 3 T2DM), verified by the last author. These statements emphasised what participants can or must be able to do to be included. Of these, six referred to language skills being sufficient to undertake RCT-related tasks [27, 36, 41, 43, 44, 46], one focused solely on “ability to speak English” [48], and the rest referred to providing consent [52–54] or completing assessments [29]. We identified 26 instances of negatively framed eligibility criteria, referring to insufficient ability, difficulties, skills deficits, or exclusion, from 12/23 depression RCTs [28, 30, 33, 34, 36, 38, 40, 42, 45, 47–49] and 3/9 T2DM RCTs [51, 55, 57]. This included reference to participants’ inability to communicate [47], use specified language skills (read/write/verbal/understand) [34, 45, 57], complete questionnaires [28, 30, 42, 55], or provide consent [36, 38, 45, 51, 57]. Two of these studies made reference to disabilities [48] or impairments [47], and 1/15 explicitly cited a “language barrier” [55].

#### Language-related accommodations

We also examined any accommodations or procedural adaptations to support participation in languages other than English. Only depression RCT recorded participants’ language preference [44]. In its observational feasibility study, the authors required either “sufficiently good English” to engage with the intervention, or “willing[ness] to work with an NHS translator”. However, because the eligibility criteria were not reiterated for the subsequent pilot RCT, it is unclear whether this translator allowance also applied to the trial component or if its omission was simply a reporting artefact. Another depression study stated in parenthesis immediately following the language eligibility requirement that “two women whose first language was not English had some language assistance in completing the assessments and/or listening visit intervention” [27]. However, the authors later clarified that these women should have been excluded due to language, as the study as designed required participants to independently complete questionnaires and engage in active listening during the counselling intervention. Eight additional prospective participants were excluded for having “poor English”. Similarly, one depression pilot RCT reported that “only English speakers were offered the intervention” (BA), citing complexities in delivering the intervention in other languages [47]. The T2DM prevention study that specifically sought to recruit South Asians reported “various changes to [trial] documents, mainly because interpreters and translation will no longer be used” as a substantial protocol amendment in an appendix to the monograph [57]. However, no explanation for withdrawing these planned language services was given. No other studies reported providing translation, interpreting, cultural adaptation, or bi- or multilingual personnel.

However, some studies had provisions for participants with disabilities or cognitive impairment. For depression RCTs, this included reading study information aloud [41, 47], paraphrasing or explaining points that were difficult to understand [48], allowing patients who were physically unable to sign their consent form due to a stroke to make a mark [41], or supporting understanding through using simple sentence structures [47], typographical enhancement [37, 39, 47], visual aids [38, 47], or gestures/action [47]. One T2DM RCT that recruited patients with learning disabilities allowed consent to be either verbal or written and co-developed accessible study materials with disabled community members [52]. Such accommodations may have benefited participants from diverse language backgrounds, but were primarily intended to help people with intellectual, physical, or learning disabilities.

#### How is language-related eligibility operationalised in the identified RCTs

We coded the eligibility criteria into eight categories to capture their functional orientation, as shown in Additional file 4. The most comprehensive eligibility statements contained three elements: (a) the language or communication skills required, (b) their relevance to the intervention or research assessment, and (c) how language-based determinations would be made (i.e. by whom and/or using what tool). Three depression studies only were in that category. One of these studies relied on clinical staff’s evaluative judgments about “sufficient understanding”, although no method for determining sufficiency was described [46]. As noted earlier, the other two studies employed standardised assessment tools for screening [36, 47]. Subsequent categories contained just elements `a' and `b' (i.e. without language screening operationalisation details) or, in the most basic cases, either one or the other. A further category was for assessments of cognitive function that used language-dependent methods without reference to language, communication, or RCT tasks. A final category was for GPs’ discretion to exclude patients they “considered… inappropriate to invite” [30] or “unsuitable to take part” for any reason [49], including potentially due to language.

### Linking eligibility statements to language demands of the intervention and primary outcome measure

Among 15/32 studies that explicitly included language screening criteria, 12 had interventions with high or medium language demands. Table 3 shows intervention coding for language demands by condition when pooling both explicit and implicit language-related eligibility criteria.

**Table 3:** Intervention coding for language demands by condition when pooling both explicit and implicit language-related eligibility criteria

Language demands of the intervention	Depression RCTs	T2DM RCTs
High	[27, 30, 33, 34, 38, 40, 43, 46]	None
Medium	[28, 41, 44, 45, 47, 48]	[52, 57]
Low	[29, 36, 42, 49]	[51, 53, 55]

Notably, 4/8 RCTs that we coded medium targeted people with learning disabilities [41, 52] or intellectual or cognitive impairments [47, 48] with BA, befriending, or nurse-supported self-management interventions. Three depression RCTs with interventions coded medium [31, 32, 35] and two T2DM RCTs coded low [56, 58] made no reference to language or communication anywhere in the monograph.

Some studies coded for high language demands of interventions referenced some language skills while overlooking others. For example, a psychotherapy-based depression study highlighted speaking in the recruitment criteria without mentioning reading or writing despite extensive questionnaire use [43]. Another RCT excluded those “not able to read and write in English” for cognitive behavioural therapy delivered via an app [34] without acknowledging a role for listening or speaking despite also using oral questionnaires. Another emphasised “understanding of English” [46], which implies receptive skills but overlooks the verbal expression necessary for a diagnostic interview and talking therapy.

All but one depression study (22/23) used standardised self-report measures as the primary outcome (questionnaires, clinical interviews). In contrast, the T2DM studies relied exclusively on non-language-based outcomes (e.g. cardiovascular or cancer events, mortality, blood sugar biomarkers, ophthalmologic assessments, physical activity). The sole exception for depression was the electroconvulsive therapy ketamine trial for severely depressed patients, with low language demands of the intervention [36]. Its primary outcome measure was a delayed verbal recall test (HVLT-R), where retrieving lexical items was central. Unlike other depression studies, where language was the medium for collecting outcome data, this study used language as the outcome itself or object of the assessment. However, English language proficiency was a potential confound for lexical recall and retention. Notably, participants in this study were screened using the Wechsler Test of Adult Reading (WTAR), an intellectual functioning test with age-adjusted scores that “uses vocabulary level as a correlate of IQ”. Participants were required to pronounce increasingly difficult irregular English words, terminating after 12 consecutive errors [43], likely disadvantaging non-native English speakers. Two other studies that recruited participants with intellectual or learning disabilities [41, 48] for BA and befriending interventions, respectively (both coded medium language demands), screened participants using the Wechsler Abbreviated Scale of Intelligence (WASI-II). Although less demanding than WTAR, the vocabulary and similarity subtests require nuanced lexical expression and abstract reasoning [60]. Lower scores on these tests may reflect limited language proficiency rather than cognitive impairment, potentially excluding linguistic minorities from participating.

## Discussion

### Operationalisation of language-related eligibility

Language-related eligibility criteria may exclude large segments of the population, even inadvertently. Systematic underrepresentation of ethnically diverse patients undermines external validity and perpetuates health inequities. By exposing whether and how language-related eligibility is determined, this UK-based systematic review of 32 extended research reports highlights a critical methodological gap that must be addressed to ensure fairer, more inclusive trials. Half of included RCTs (57% depression; 33% T2DM) explicitly referenced language in their eligibility criteria, with a higher proportion for interventions where language is integral (e.g. talking therapies, collaborative care) than where it is not (e.g. pharmacological trials). Taken together, depression RCTs, which generally entail higher language demands, placed greater emphasis on language and provided more extensive, varied, nuanced, and language-centred eligibility descriptors than the T2DM trials, which largely focused on informed consent.

The proportion of T2DM trials explicitly referencing language eligibility in this study (33%) roughly aligns with 23% in UK cardiovascular trials protocols [15] but is lower than the 50% in mostly US-based T2DM trials reported in journal articles [11]. This difference is perhaps unsurprising given longstanding American legislation mandating the inclusion of ethnic minorities in clinical research coupled with language considerations that often accompany ethnicity. However, the impact of such legislation is complex. While these mandates exist, current data suggest that significant gaps in minority inclusion persist in the USA [61], which is also substantially more ethnically diverse than the UK [62]. In short, legislation is only one of several factors that likely contributes to differences across contexts rather than the sole driver.

As in the previous systematic reviews [11, 15], language proficiency was not consistently operationalised across studies. We extended these insights by systematically coding the language demands of interventions and primary outcome measures. Some trials required language skills that were not reflected in eligibility criteria, while others employed language-based primary outcomes but failed to directly mention or indirectly invoke language in their eligibility statements. In such cases, language requirements were underspecified or treated as a stealth factor, unacknowledged and potentially subject to inconsistent interpretation by recruiters despite being integral to participants’ engagement with trial procedures. Vague and inconsistent language screening risks excluding ethnically diverse participants and reinforcing systemic barriers.

Unlike the previous systematic reviews, this study identified insights on how language gatekeeping was operationalised during recruitment for those depression studies reporting this detail. Two RCTs used standardised assessment tools but risked either conflating limited English proficiency (LEP) with communication disorders, or underestimating cognitive ability in linguistic minorities. Without accommodations or validated alternatives, such practices could introduce bias and systematically exclude linguistically diverse participants.

To mitigate this, a purpose-built, standardised language screening tool could help ensure fair and consistent eligibility decisions if applied uniformly to all participants. If participation is restricted to native English speakers for principled reasons (i.e. absence of validated measures and not because language accommodations are deprioritised in the budget), then a standardised tool could help ensure that exclusions are transparent, consistent, and grounded in construct-relevant criteria. The development and validation of such a tool would be best undertaken through a collaborative, interdisciplinary process. Language assessment researchers would be well placed to lead the design and validation of the instrument given their joint expertise in linguistics and psychometrics. Input from clinical trial methodologists, researchers involved in participant recruitment, and medical specialists with expertise in specific conditions would be essential to ensure that the tool is feasible, appropriate, and aligned with trial workflows. PPIE contributors would also play a crucial role in providing insight into acceptability and burden from a participant perspective. Representatives from all groups should be involved from the outset. The responsibility for commissioning and coordinating the development of such a tool could lie with funders or research infrastructure bodies, who are well positioned to support standardisation efforts across trials for a given condition or intervention type. We consider a single universal tool to be infeasible, given the variability in trial populations and language-related trial demands. In sum, we do not propose that individual trial teams develop bespoke instruments, but rather that a sector-wide, multidisciplinary initiative would be desirable to ensure methodological rigour, usability, and, through rigorous piloting and field testing, broad applicability.

Most trials relied on subjective recruiter judgments rather than formal assessment tools. For example, some studies required sufficient language skills without defining what constituted sufficiency or how it would be assessed. In one depression study [49], inconsistent phrasing of eligibility as “unable to complete” or “could not understand” questionnaire items in different parts of the monograph suggests confusion about the construct and interpretive liberties in applying the criteria. This underscores the need for clarity regarding both the way that language-related eligibility is framed, and the methods used to assess it, not only for research reporting, but also for trial staff conducting screening. In another depression trial, conditional phrasing (“if…would”) implied exclusions without giving participants the opportunity to attempt questionnaire completion. Relatedly, two depression studies [30, 49] granted GPs unrestricted discretion to exclude participants for non-clinical reasons, introducing variability and potential bias. Such practices risk eliciting judgments about language ability that are disconnected from participants’ actual performance on trial-relevant tasks. For example, reverse linguistic stereotyping may occur when assumptions about a person’s language ability are based on their phenotype or (perceived) ethnicity [12]. Similarly, recruiters may mistake a foreign accent for unintelligibility even when the speaker is fully capable of engaging with the intervention [13].

Notably, the depression trials recruiting participants with cognitive or learning disabilities had the most comprehensive language-related eligibility criteria. This suggests more purposeful language screening when language is medically salient than when it is not. Alongside the one T2DM study recruiting participants with (learning) disabilities, two further T2DM RCTs that framed language eligibility explicitly without resorting to generic statements about consent recruited >20% of ethnic minorities, among the highest in the review. However, it remains unclear whether a stronger focus on language facilitated more diverse recruitment or whether language considerations were primarily introduced in studies aiming for greater ethnic representation. This trend warrants confirmation in future research.

About a quarter of depression studies and one T2DM RCT described the language skills needed to complete a research task, yet none specified how this would be assessed. These descriptions reflect a functional approach to language assessment, whereby performance descriptors focus on what participants can do [59] at the level implicitly deemed necessary for trial participation. Making task requirements explicit through functional “can do”-style descriptors can shift emphasis away from vague proficiency labels toward the concrete communicative demands of participation. However, >60% of these statements were negatively framed, emphasising tasks participants were assumed to be unable to perform, were judged to lack capacity for, or were unwilling to undertake. This reflects a deficit framing [63], implicitly placing responsibility on participants considered unable to engage in English as required, with little evidence of effort from trial teams to offer accommodations.

While some trials adapted materials or procedures for participants with disabilities, none provided language support or cultural tailoring for people from different language backgrounds. Two depression RCTs [27, 47] explicitly acknowledged methodological constraints that precluded linguistic minorities from participating, disclosing that the trials were not designed to include such groups. The latter study suggested that, if proven effective for “English speakers”, the intervention should later be extended to “non-English speakers” but cautioned that translated versions may diverge from the original, potentially resulting in non-equivalent interventions [47]. This underscores the need to distinguish between interventions where language is integral and those where it is not. In the former, changing the language medium could render the English original and translated versions incomparable, whereas making language accommodations for trials where language is not central (e.g. dosing trials) is unlikely to distort the intervention. Determining optimal translation and interpreting methods to enable broader participant inclusion is fertile ground for future research [64], particularly for conditions like depression, which may be stigmatised in some cultures and lack direct lexical equivalents in certain languages [19]. A recent study obtained input from “racially marginalised community groups” on patient information leaflets reportedly designed to be more inclusive, resulting in a framework of 74 practical recommendations for improving accessibility [65]. These recommendations span broad categories such as formatting, information structures and volume, plain and inclusive language, visual aids, with the potential for non-written formats, and co-production with diverse communities. Such approaches to enhancing the accessibility and inclusivity of patient-facing materials are promising, though it is important to recognise that “marginalised communities” are not a monolith, with further research needed on linguistic and cultural tailoring for different groups.

### Diversity of the recruited sample and research reporting

Foregrounding these results is the staggering lack of ethnic diversity in the depression RCTs, which recruited just over 3% of non-White participants as a proportion of the recruited sample. This finding suggests that research teams are not doing enough to achieve a more diverse and representative sample and that certain methodological aspects of the trials likely pose barriers to wider participation. Trial teams would benefit from using prospective tools such as the INCLUDE Ethnicity Framework [8], which was developed to support researchers identify and mitigate potential sources of exclusion to promote more inclusive and equitable trial design, with some funders now signposting to this tool. However, we note this resource was not yet available when these studies were undertaken.

The included T2DM RCTs present a mixed picture. A third almost exclusively recruited White participants, another third recruited a more diverse sample, and the remaining third did not report ethnicity data. According to the 2025 CONSORT guideline 25, “Baseline data”, participant demographic variables, including “race and/or ethnicity, culture and/or religion, language…”, should be presented in tabular format alongside clinical data [25]. The inclusion of language is notable, underscoring that it should not be conflated with race or ethnicity. For example, census categories in England and Wales such as “White other” or “Pakistani” provide no information about whether participants are monolingual English speakers, bilingual or multilingual, or have LEP. To improve research reporting, trials should record participants’ preferred language and offer bilingual options where feasible [66].

Journals also have a role to play in improving reporting standards (and, hence, the data collected) by requiring demographic reporting in published work, as exemplified by *Trials*’ mandatory protocol and trial results reporting requirements [67]. A systematic review of 167 articles on acute strokes in the USA, of which only 9% were trials, found that 10% of included studies explicitly reported including participants with LEP in the article, dataset, or registry [68] compared to 8% in the US population estimate, whereas 87% did not feature language as an eligibility criterion. LEP in this context refers to participants aged ≥5 years who reportedly speak English “less than ‘very well’” based on US Census data [69]. UK-based trials should adopt a standard approach to measuring and reporting language variables, at a minimum aligning with census question formats (e.g. main language, self-reported proficiency). Collecting and reporting such information is crucial to understanding recruitment and retention barriers within and across ethnic groups, assessing external validity, and accommodating participant language preference where feasible. Studies and research support services should budget for translation and interpreting, potentially using multimodal formats [65], and consider hiring multilingual, multicompetent staff [14].

Notably, second language attainment is shaped by multiple factors that trials rarely record, including the quality and intensity of language input, opportunities for meaningful exposure and use, and the age and context of language acquisition [70]. In our dataset, 57% of depression studies and just 1/9 T2DM study reported participants’ educational attainment or qualifications, although with inconsistent categories and granularity. Recent research demonstrates that years of schooling robustly predicts second language test performance among adult migrants with limited formal education or low literacy levels [71]. This suggests that educational attainment should be routinely captured and reported in trials alongside other baseline participant characteristics [25] with usable descriptive categories. For example, a study capturing whether participants have UK upper secondary qualifications (A-levels) or above as a categorical variable [27, 28, 30, 40] is likely to be less applicable to migrants and understandable to internationally educated participants than a study capturing whether participants left school before or at a given age [43]. In short, educational attainment warrants further investigation in future trials research on language.

### Limitations

This systematic review has several limitations. First, during write-up, we identified indexing issues with the NIHR Research Journal’s “Primary research” search parameter, which likely resulted in fewer studies being retrieved than should have been included. This, combined with the review’s timespan not encompassing the service’s full tenure (the service was discontinued in 2025, whereas the review period ended in early 2022) means that our review is less comprehensive than intended and may not proportionately reflect trials across all NIHR funding streams (e.g. Programme Grants were retrospectively found to have been underrepresented). The 2010 starting point was applied to maintain feasibility given limited funding and to focus on contemporary RCT reporting, but we acknowledge that this cut-off was somewhat arbitrary. We also acknowledge the time elapsed since the search and that the growing recognition for research diversity and inclusion would not have been reflected in this evidence synthesis. However, we do not believe that this truncation and search engine limitations materially affected the findings. Second, given the NIHR’s shift away from monograph publication, the direct applicability of our findings to future trial reporting may be constrained. Notwithstanding this, we consider the monograph-based snapshot valuable precisely because it documents practices under a detailed reporting regime and preserves a historical record of reporting conventions that may inform future standards. Moreover, comprehensive reporting is still feasible outside monograph formats. Many journals now offer flexible or uncapped word limits, established reporting guidelines (e.g. CONSORT) are agnostic to publication type [25], and the use of monographs persists within the clinical research ecosystem (e.g. Clinical Study Reports) [72]. Any transition away from more detailed reporting places greater responsibility on journal editors and peer reviewers to reinforce clear eligibility reporting within guidelines and to encourage public archiving of protocols and supplementary materials in open repositories so that methodological detail remains accessible.

Third, numerous research instruments were used for baseline or participant screening and/or primary outcome assessments, in which language was either the object of assessment or, more commonly, the medium through which assessments were conducted (e.g. self-report measures). A detailed analysis of the language demands of these instruments and procedures, including whether they had been validated in languages other than English, was beyond this study’s scope, which was limited to what monograph authors reported. In-depth examination of those tools would be crucial for nuanced identification of how instruments and procedures may pose barriers to individuals and groups, plus mitigation strategies. We encourage trial teams to undertake such analyses, ideally while consulting relevant tools to interrogate their appropriateness and consider the potential effects of their use when designing studies. Fourth, as language experts, the first and second authors coded the intensity of language demands but could have corroborated their classifications with an established benchmarking tool for language performance [59].

Fifth, we did not assess the methodological quality of included studies themselves. Sixth, although we incorporated the perspectives of community organisations and PPIE members through this collaboration, it remains important to further explore the insights and experiences of linguistic minority participants. This includes their views on eligibility criteria as well as broader barriers to recruitment and retention. Equally important are the perspectives of recruiters, particularly regarding how they make language-related decisions. Their views and lived experiences could inform the future development of a standardised language-specific tool to support consistent and fair eligibility decisions, reducing reliance on gut instinct. Ideally, such tools would be tailored each condition’s or intervention type’s specific language demands (e.g. incorporating relevant lexical items): one-size-fits-all is unlikely to be effective. Any such tool should be applied to all participants, as selectively administering language screening based on participants’ name, skin colour, or accent would introduce additional barriers and raise ethical concerns. Technological advances also offer new possibilities for providing linguistic accommodations. However, while artificial intelligence (AI) tools may serve as a useful first step, they cannot yet replace the nuanced understanding and accuracy provided by human translation and interpreting [73]. Finally, we recognise that restricting the review to clinical depression and T2DM necessarily excludes other health conditions disproportionately affecting ethnic minority groups that are also important for understanding equity and participation in trials.

## Conclusions

Language considerations are integral to diversity and inclusion strategies, for recruitment and retention, by health funders, regulators, and multinational pharmaceutical companies. Key recommendations include (a) reducing language barriers by implementing language-related accommodations to support more diverse participant recruitment and improve retention; (b) critically examining language-related exclusions during recruitment, including the wording of eligibility criteria (e.g. avoiding deficit framing); (c) ensuring alignment between these criteria and each trial’s language demands, while also reflecting on the fairness and consistency of language-based screening across baseline assessments, interventions, and outcome measures; and (d) systematically collecting participants’ language data alongside other demographic variables to adequately describe the sample and disentangle language from race or ethnicity. Doing so while also incorporating stakeholders’ perspectives will enhance our understanding of the drivers of diverse participation.

## Supplementary Information

Additional file 1.

Additional file 2.

Additional file 3.

Additional file 4.

Additional file 5.

## Data Availability

Secondary data supporting the conclusions of this article and supplementary material are included within the article and its additional files and are available on the Open Science Framework (OSF): https://osf.io/eshcb/overview.

## Abbreviations

BA: Behavioural activation
CONSORT: Consolidated Standards of Reporting Trials
GP: General practitioner
HTA: Health Technology Assessment
HVLT-R: Hopkins Verbal Learning Task—Revised
PROSPERO: International Prospective Register of Systematic Reviews
LEP: Limited English proficiency
MRC: Medical Research Council
NHS: National Health Service
NIHR: National Institute for Health and Care Research
PPIE: Patient and public involvement and engagement
PRISMA: Preferred Reporting Items for Systematic Reviews and Meta-Analyses
RCT: Randomised controlled trial
T2DM: Type 2 diabetes mellitus
WTAR: Wechsler Test of Adult Reading
