# Supplementary material for: Language-related eligibility criteria in UK randomised trials: a systematic review of extended research reports
Source: Trials. 2026 May 16;27:476. doi: 10.1186/s13063-026-09766-5 (PMC13348756; doi:10.1186/s13063-026-09766-5)
Supplement: Supplementary file 2 — Additional file 2. [file 13063_2026_9766_MOESM2_ESM.pdf]

Additional file 2: Data extraction form

**Variables**	**Coding notes or categorisations**
NIHR report metadata	
Search date	Sept. 2021; Feb. 2022
Main search term	Depression; Diabetes
Manuscript ID	Accession number used as file identification number
Title of NIHR report	
First author's surname	
All authors	
Abstract	
Year of publication	
NIHR journal that the report was published in	EME (Efficacy and Mechanism Evaluation)<br>HDSR (Health and Social Care Delivery Research)<br>HTA (Health Technology Assessment)<br>PHR (Public Health Research)
RCT characteristics	
Trial sites	Single- or multi-centre
Randomisation	Individual, cluster
Arms	No. of arms of the trial
RCT design	Authors' description<br>May refer to elements such as number of trial arms, single/multi-centre, phase, individually/cluster randomised, pragmatic/adaptive/prevention trial, etc.
Self-described as a pragmatic trial?	Yes or no
Trial setting	Pilot, feasibility, full trial
Medical condition(s) targeted	Depression, diabetes, and any further description
Brief description of the control condition	
Characteristics of the recruited patient sample	
Number of patients recruited to the RCT	Note that in some instances, we needed to compute the total sample size based on the authors' reported sample size for each group or arm of the trial
Age	Note that most but not all studies reported means and either SDs or ranges. Where possible, we aggregated group statistics (i.e., by trial arm) to report indices for the whole sample
Sex/gender	We report the variables and categories that the authors used
Ethnicity	We report the categories that the RCT authors used
Sociodemographic variables	E.g., education level, employment status, marital status
First or second language literacy	Any measure of literacy or description of assessing it
Health literacy	Any measure of health literacy or description of assessing it
Other patient-related variables	E.g., cognitive function, access to technology
Control condition	
Brief description	
Person administering the control condition	
Tailoring of the control condition to individuals/groups	Any tailoring reported
Intervention	

*Description of the intervention	
*Why	Rationale, theory, or goal of the elements essential to the intervention
*What	Physical or informational materials used in the intervention
*What	Procedures, activities, and/or processes used in the intervention
*Who provided	Intervention provider
*How	Modes of delivery. E.g., face-to-face, web, oral [tablets], injections
*Where	Location of intervention
*When and how much	Frequency and duration of the intervention
Language skill(s) required to receive the intervention	0 = None<br>1= Listening<br>2 = Reading<br>3= Speaking<br>4 = Writing
Technologies involved in receiving the intervention	1= Phone<br>2 = Web/app
Coded language/communication demands of the intervention for patients	Low/Medium/High
Outcomes	
Primary outcome	E.g., a specific questionnaire or clinical measure
Language/communication demands of the primary outcome measure	Low/High
Coded language demands of primary outcomes.	1=Self-report, high<br>2=Clinical measures, low
Description of secondary outcomes in terms of language demands	
Translation/interpretation/language accommodations/cultural tailoring	
Access to translation and/or interpretation services or any additional language-related accommodations or cultural tailoring	Searched manuscript for any reference to accommodations/tailoring during participant screening, for the intervention, or for outcome measures
Alternate consent pathways or language-related accommodations during the informed consent process (informed written consent unless otherwise stated)	
Available language(s) of patient-facing materials and other communications	
People/tools supporting language accommodations	
Recruitment, eligibility criteria, and other language information	
Recruitment details, including language-related participant screening and baseline assessments before randomisation	
Eligibility criteria explicitly mentions language	
Eligibility criteria refers to patients needing to conduct a task that involves language use but without directly referring to language	
Eligibility criteria where language *may* play a role in gatekeeping	
Direct reference to language and human judgements or standardised assessments in eligibility decisions	
Language eligibility directly referred	1 = Yes, 0 = No

Eligibility criteria where language may play a role in gatekeeping	
Other patient eligibility criteria	Clinical and nonclinical eligibility criteria that bear no relation to language/communication
Additional information about language	

*These data extraction categories were derived from the TIDieR checklist on reporting details about interventions.

Tammy CH, Paul PG, Isabelle B, Ruairidh M, Rafael P, David M, et al. Better reporting of interventions: template for intervention description and replication (TIDieR) checklist and guide. BMJ: British Medical Journal. 2014;348:g1687.
