# Supplementary material for: Language-related eligibility criteria in UK randomised trials: a systematic review of extended research reports
Source: Trials. 2026 May 16;27:476. doi: 10.1186/s13063-026-09766-5 (PMC13348756; doi:10.1186/s13063-026-09766-5)
Supplement: Supplementary file 4 — Additional file 4. [file 13063_2026_9766_MOESM4_ESM.pdf]

Additional file 4: Coding scheme and coded categories of included studies' eligibility statements

Legend
+ positive framing of eligibility criterion (i.e., what participants can do sufficiently)
– negative framing of eligibility criterion (i.e., what participants cannot do sufficiently)
== same or very similar semantic meaning to another eligibility criterion from the same study (i.e., no change in or addition of major content words, although the nuance may slightly change)
? unable to code whether eligibility criterion is positive or negative
⊕ Reference to required language/communication skills
❁ Reference to RCT task(s) (e.g., research assessments, intervention, providing informed consent)
🌀 Direct reference to how skills/abilities required for participation would be assessed
DEP: Depression RCT
DBT: Diabetes RCT
< >: Researcher memo – researcher observation or interpretation, sometimes in conjunction with details about the RCT from data extraction

Note that dates are listed when there are multiple RCTs with the same first author.

Note that when studies included eligibility criteria that were articulated in more than one place within the NIHR monograph (e.g., both *Scientific summary* and *Methods* section), each instance of eligibility was recorded separately so that any differences in expression between the statements, even if minor and with no bearing on meaning (e.g., "able" vs. "the ability to"), could be examined for consistency and semantic differences. Similarly, different bullet points, even if partially overlapping with previous eligibly criteria, were taken to be distinct idea units and were recorded separately, as were punctuation marks that indicated a new element of eligibility from a syntactic perspective (e.g., separate clause given in parenthesis; Sharp). This resulted in a total of 46 distinct eligibility criteria, excluding category 9 at the end of this document that notes the studies where there was an absence of any such criteria. In this document, there are between 0 and 6 eligibility statements listed per study for 32, with most only including one listing. The RCT at the upper limit of the range (Thomas, 6) and the second most frequent in terms of number of statements listed (Jahoda, 4) were both depression studies that specifically recruited stroke patients and patients with learning disabilities, respectively, and tested a behavioural activation intervention. Taken together, these studies were among the most complex or involved in terms of language-related screening. Thus, frequency may be an indicator of the complexity of the language and communication element of the study but is also a function of research reporting.

Presence of eligibility criteria that referred to or implied language or to related screening activity

1. Language/communication skills needed for intervention or research assessment, with specification of how that determination is made (i.e., specific research instrument/human judgment) ⊕❁🌀

Thomas – "had communication difficulties that would have an impact on their capacity to take part in the intervention, based on assessment with the Consent Support Tool (CST) for people with aphasia" DEP

Thomas – "had visual or hearing impairments that would have an impact on their capacity to take part in the intervention based on their therapist's opinion at baseline assessment" DEP

> [Study recruits post-stroke patients. Having visual or hearing impairments is of course different than having sufficient language/communication skills]

Anderson + "Verbal intelligence quotient (IQ) equivalent to ≥ 85 and sufficiently fluent in English to validly complete neuropsychological testing" DEP

> <Electroconvulsive therapy study; Verbal IQ assessed through Wechsler Test of Adult Reading; not clear what constitutes *valid* testing>

Serfaty + "Sufficient understanding of English judged <u>by clinic staff</u> to enable them to engage in CBT [cognitive behavioural therapy]" DEP

> <Study recruits individuals with learning disabilities; Uses Wechsler Abbreviated Scale of Intelligence>

2. Language/communication skills needed for intervention or research assessment, but how that determination is made implied and not directly given ⊕❁

2a. Language/communication skills needed for specified intervention or to complete questionnaires (i.e., some task specificity), although with how such determinations are made or who is making them not specified ⊕❁

S Richards 2018 + "Potential participants also needed to have sufficiently good English-language skills to engage with both the mental health-care co-ordination and BA [behavioural activation] components of the EPC [enhanced psychological care] intervention " DEP

> <This statement was provided in reference to the non-randomised observational feasibility study observational feasibility study with an interview component that was sequentially reported first. Not clear if it also applied to the pilot RCT, although we have included it with the assumption that it did and that not repeating the same information was an artifact of research reporting.>

Ali – "Individuals with intellectual disability were excluded if they had limited communication and comprehension skills that would prevent completion of the questionnaires" DEP

<The use of conditional in the framing suggests that participants may have been excluded without being given the opportunity to complete the questionnaires, which could introduce bias). Also unclear how sufficiency is determined. No cut-off provided and no indication of who makes that judgment>

> <All of these statements have two clauses>
>
> <Not clear if patients given the opportunity to demonstrate this, as also uses the conditional. However, elsewhere in RCT reporting, there is specific mention of how determinations about language ability are made using a specific tool>

Jahoda + "sufficient communicative abilities to engage in therapy" DEP

2b. Language/communication skills needed for generically framed research assessments, the intervention, or to provide informed consent, although with how such determinations are made or who is making them not specified ⊕❁

Jahoda + "Had a level of expressive and receptive communication skills in English to allow for participation in treatment (reading skills were not required)" DEP

Thomas – "had communication difficulties that would have had an impact on their ability to take part in the intervention" DEP

> <Makes an assumption about an impact that participants' communication difficulties would have if they were to take part in the intervention, although not given an opportunity. Written using the conditional (hypothetical)>

Thomas – "had communication difficulties that would have had an impact on their ability to take part in the intervention" DEP

Lynch +"patients had to [...] speak English well enough to participate in the treatment and the study" DEP

Sharp + "were proficient in English at a level to complete all research assessments" DEP

House + "provided written or verbal (when necessary) informed consent" DBT

> <This past tense framing was because participants needed to have first consented to participate in an earlier non-randomised phase of the study to be eligible for the subsequent feasibility RCT, for which this criterion was listed>

2c. Inability to complete/understand questionnaires in light of language barrier, though how exactly that determination is made implied and not given ⊕❁

<“In English” was interpreted as direct reference to language/communication skills needed to participate>

==Chalder – "unable to complete self-administered questionnaires in English” DEP

==Chalder – "inability to complete self-administered questionnaires in English" DEP

Duffy – "were unable to complete self-administered questionnaires in English" DEP

Duffy –"could not understand questionnaires in English" DEP

<Lack of consistency in how language ability is framed at different points in Duffy’s report>

Ruban – "Language barrier, mental incapacity, unwillingness or inability to understand and be able to complete questionnaires" DBT

<Lumped together in a list>

3. Assessment of cognitive function made using language-dependent methods, though without direct reference to language/communication skills or RCT task requirements 🌀

Jahoda - "mild to moderate learning disabilities" (p. xxvi), as assessed using the Wechsler Abbreviated Scale of Intelligence (WASI)” DEP

D Richards 2017 – “People who were… cognitively impaired, ascertained at baseline by research interviews” DEP

House - “mild to moderate learning disability, identified by GP on clinical assessment and confirmed by researcher on the basis of functional history and performance at interview” DBT

4. Reference to language/communication skills but not linked to RCT task requirements, all with negative framing ⊕

Ali + "ability to speak English" DEP

==Thomas – “are unable to communicate in English” DEP

==Thomas – “were unable to communicate in English” DEP

Thomas – “were unable to communicate in English prior to the stroke” DEP

<Only the third statement specifies that this applies to “prior to the stroke.” Therefore, the recruiter could potentially exclude people by reading medical notes from before the stroke>

Littlewood – "They were not able to read and write in English" DEP

Brabyn – "not able to read and write in English" DEP

Khunti – "unable to understand basic written and verbal English" DBT

Burroughs – "inability to understand or read English" DEP

5. No reference to language/communication skills when referring to (in)ability to complete research assessments, including questionnaires ❁

Bedson + “able to complete the research assessments” DEP

Wiles – “patients who were not able to complete the study questionnaires” DEP

Kessler – "patients who were unable to complete the questionnaires" DEP

6. No reference language/communication skills when referring to ability/capacity/willingness to provide informed consent ❁

S Richards 2018 + "Potential participants also needed… to provide informed consent to take part" DEP

Jahoda + "the ability to provide informed consent" DEP

Bedson + "able to give informed consent" DEP

Sivaprasad 2018 + "Ability to give informed consent" DBT

Anderson + "Capacity to give informed consent" DEP

> <Electroconvulsive therapy study for people with major depressive episode due to unipolor or bipolar disorder. Used Wechsler Test of Adult Reading to test cognitive function>

==Khunti – "unable to provide informed consent" DBT

==Khunti – "unable to give informed consent" DBT

Anderson – "Unable to give informed consent" DEP

Griffin – "unable to provide written informed consent" DBT

Gabbay – "were unable or unwilling to give written informed consent to participate in study" DEP

Burroughs – "lacking capacity to consent" DEP

> <Study involving older people that assessed cognitive capacity assessed as part of participant screening>

Sivaprasad 2019 +" Ability to give informed consent throughout the duration of the study" DBT

7. Availability of help to complete tasks as part of eligibility criteria

Sharp ? "(two women whose first language was not English had some language assistance in completing the assessments and/or listening visit intervention)" DEP

> <Tagged onto the end of the eligibility criterion, "were proficient in English at a level to complete all research assessments." But the authors later divulged in the Discussion that "there were instances where women were not excluded who should have been (for example the two women whose English language skills should probably have made them ineligible) (p. 87). They also acknowledged that "the study as designed was unable to include women for whom English was not a first language or at least for whom completion of the questionnaires unaided or participation in listening visits would not be possible" (p. 90)>

S Richards 2018 + "... or to be willing to work with a NHS [National Health Service] translator if required" DEP

> <This statement was provided in reference to the non-randomised observational feasibility study with an interview component that was sequentially reported first. Not clear if it also applied to the pilot RCT, which did not list its own eligibility criteria and, instead, drew on that from the earlier study. Therefore, we have included it with the assumption that this also applied to the RCT component, alongside the other eligibility criteria.>

> <Other studies did provide language related support (e.g., research assistant to facilitate participant understanding of "treatment principles" in Ali, but not reported as part of eligibility criteria)>

8. GP recruiters given license to exclude based on their discretion

> <These studies were not included in counts relating to language-related eligibility criteria, because they relate to GP discretion about inclusion but do not *necessarily* involve language-based judgments. In addition, language is not the object of the task or assessment>

Wiles – "GPs excluded any patients whom they considered it would be inappropriate to invite" DEP

Duffy – "The GP could also decide that the person was unsuitable to take part in the trial on any other grounds" DEP

<One reasons for these exclusions could be assumptions about language>

<u>**Absence of criteria that referred to or implied language or to related screening activity**</u>

9. No mention of language or using language as a proxy in eligibility criteria

9a. Although not among the listed eligibility criteria, the RCTs did report how they operationalised language screening checks elsewhere in the monograph

Lewis: 99.3% White, Medium Collaborative care, baseline self-report questionnaires, phone screening diagnostic interview DEP

<No language-related recruitment criteria stated. However, the following information was divulged in the report [for older participants who may have cognitive impairment]: “Questions [during the baseline assessments] were also added to help case managers assess participants’ understanding of the treatment principles">

Bosanquet: 98.5% White, Medium Collaborative care, baseline self-report questionnaires DEP

<No language-related recruitment criteria stated, as for the Lewis study. However, the following information was divulged in the report, mirroring almost verbatim what was reported in Lewis: “Questions were also added to help the case manager assess the participant’s understanding of the treatment principles">

<Note that both of these studies (CASPER and CASPER Plus trials, which were published in the same year, used the same research design and intervention but targeted different populations (subthreshold depression vs major depression, respectively)>

9b. No indication that language was a factor that may have been considered in eligibility screening anywhere in the report

Kuyken: 99.1% White, Medium mindfulness based cognitive therapy group sessions, structured clinical interview primary outcome measure DEP

Ferrier: 92.5% White, Low drug trial, depression self-ratings primary outcome measure DEP

D Richards 2016: Cluster randomised, 85% White, Medium Collaborative care, PHQ-9 written self-report questionnaire primary outcome measure DEP

Hykin: No reporting of ethnicity, Low Drug trial DBT

Miras: No breakdown of ethnic composition of recruited sample: “"The majority of the patients were middle-aged, white, European and female.” Low Surgical trial DBT

Language as a stealth factor. For Kyuken and Richards 2016, language is inherent to the intervention, so the lack of mention of it is glaring.
